# Transcriptomic and physiological analyses reveal that cytokinin is involved in the compound leaf development of alfalfa

**DOI:** 10.3389/fpls.2025.1460205

**Published:** 2025-01-29

**Authors:** Hongyao Mei, Jiajun Yan, Xuexin Jia, Weilin Wang, Shuangshuang Li, Ruiqi Sun, Hongjiao Jiang, Lijun Xie, Chuanen Zhou, Shiqie Bai, Lu Han

**Affiliations:** ^1^ The Key Laboratory of Plant Development and Environmental Adaptation Biology, Ministry of Education, School of Life Sciences, Shandong University, Qingdao, China; ^2^ School of Life Science and Engineering, Southwest University of Science and Technology, Mianyang, China

**Keywords:** alfalfa, compound leaf development, cytokinin, forage quality, KNOX1

## Abstract

Alfalfa is one of the primary forages, and its yield is largely dependent on the development of its leaf. In this study, to elucidate the mechanism of compound leaf development, we isolated and examined the alfalfa phenotype Chuancao No.7, exhibiting five leaflets. The agronomic traits of the Chuancao No.7 including the leaf blade area, leaf/stem ratio, total fresh weight, and dry weight showed significant increases compared to those of the wild-type. Analyses of forage quality traits indicated significant differences in crude protein (CP), acid detergent fiber (ADF), crude fat (CF), water-soluble sugars (WSS), carbon content, ash composition content, and phosphorus content between Chuancao No.7 alfalfa and wild-type. Transcriptomic profile analysis revealed that differentially expressed genes were identified in the cytokinin (CK) signaling pathway. Both exogenous treatment and endogenous CK content detection indicated that cytokinin played a key role in the development of the alfalfa compound leaf. These results serve as a valuable resource for optimizing the forage quality and exploring the excellent germplasm of alfalfa.

## 1 Introduction

Alfalfa (*Medicago sativa* L.) is a leguminous forage renowned for its high quality and yield worldwide (Wang et al., 2024). Owing to the alfalfa’s high protein content, vitamins, and minerals content, the crop is an excellent nutritious forage for ruminants (Hojilla-Evangelista et al., 2017). One of the main goals of alfalfa breeding is the improvement of forage biomass which is closely associated with the relative production of the leaf (Jungers et al., 2020). Mature leaves of alfalfa have a three-pinnately compound leaf pattern (Zhang et al., 2021). Leaves are initiated from the peripheral zone of the shoot apical meristem (SAM), where stem cells self-renew and produce leaf primordia (Ge et al., 2010). The conversion of leaf primordium into mature leaf results from the strict coordination of cell proliferation and differentiation. A complex regulatory network, which requires multiple transcription factors and phytohormones, is involved in both simple and compound leaf development (Bar and Ori, 2015).

The *class KNOTTED I-like homeobox* (*KNOXI*) genes play important roles in leaf development in many species, particularly in most compound leaf species. They promote and maintain the activity of the SAM by activating the biosynthesis of cytokinin (CK) (Jasinski et al., 2005). The first *KNOX* gene to be identified in plants was *KNOTTED1* (*ZmKN1*) in maize. Ectopic expression of *ZmKN1* leads to inhibition of leaf differentiation (Vollbrecht et al., 1991). In *Arabidopsis thaliana*, the expression pattern of *KNOTTED1* (*KNAT1*) during vegetative development is similar to that of the *ZmKN1* maize gene (Lincoln et al., 1994). The *KNOXI* mutants in rice, *Oryza sativa homeobox1* (*osh1*) and *Oryza sativa homeobox6* (*osh6*) fail to establish the SAM both in embryogenesis and regeneration, revealing that *KNOXI* gene expression positively regulates the phytohormone CK (Tsuda et al., 2011). Both peas and soybeans produce compound leaves, but their expression patterns of *KNOXI* genes differ. *KNOXI* genes are expressed in soybean leaf primordia; in contrast, these genes are not expressed in pea leaf primordia (Champagne et al., 2007). In *Medicago truncatula* (*M. truncatula*), the expression of *MtKNOXI* was observed at the early stages of leaf primordia formation, and overexpression of TM/BP‐like *MtKNOXI* genes is sufficient for increasing leaf complexity (Peng et al., 2011; Zhou et al., 2014). Overexpression of a *KNOXI* gene in alfalfa results in an increased number of leaflets per petiole (Champagne et al., 2007). Based on the above research results, class I KNOX proteins play an essential role among both simple leaf model species and compound leaf species.

Molecular genetic studies indicate that the genetic regulation of compound leaf development is complex. Previous research has reported *LEAFY* (*LFY*) orthologs function as substitutes for *KNOXI* expression to regulate compound leaf development in legumes of IRLC clade, such as *M. truncatula* and *M. sativa* (Pautot et al., 2022). The *SINGLE LEAFLET1* (*SGL1*) is expressed in the entire SAM and plays a significant role in initiating lateral leaflet primordia (LL) during compound leaf development in *M. truncatula*. Loss-of-function mutants of *SGL1* develop simplified or simple leaves (Wang et al., 2008). The role of the *LFY* orthologs in soybean compound leaf development is similar to *SGL1*. The *GmLFY* RNAi lines produced leaves at the second node that showed reduced complexity (Champagne et al., 2007). Therefore, as the transcription factor, *LFY* plays an important role in the vegetative development of plants.

CK is an important mobile signal for SAM homeostasis and is necessary for plant leaf development processes. CK has been shown to affect a range of processes, from the regulation of the SAM to leaf senescence (Kieber and Schaller, 2018). Type-B *ARABIDOPSIS RESPONSE REGULATORS* (*ARR1*, *ARR10*, and *ARR12*), the main response genes involved in CK signaling, directly bind to the promoter and activate the transcription of the transcription factor gene *WUSCHEL* (*WUS*) (Lindsay et al., 2006). In *M. truncatula*, a WUSCHEL-like homeobox family transcriptional regulator *STENOFOLIA* (*STF*) regulates leaf blade expansion through the CK pathway (Tadege et al., 2011). *ARABIDOPSIS HISTIDINE KINASE 2* (*AHK2*), and *ARABIDOPSIS HISTIDINE KINASE 3* (*AHK3*) as CK receptor genes have a prominent role in the control of leaf development. The *ahk2 ahk3* double mutants developed fewer cells in leaves, and reduced chlorophyll content (Riefler et al., 2006). These studies reveal a complex regulator network of CK and interactions within different gene families in leaf development.

Leaves are the primary photosynthetic organs in plants and serve as a significant constituent of forage. Significantly advancements have been achieved in the research on compound leaf development among various species (Wang et al., 2008, 2013; Bar and Ori, 2015). However, our research progress on compound leaf development in alfalfa remains limited. This study developed a new alfalfa variety, characterized by its multiple leaflets of compound pattern derived from the parent Algonquin population. The new variety was identified based on a specific criterion more than 70% of the multi-leaflet forms of each plant. So, it has been officially registered in China as Chuancao No.7. Our research focused on the characteristics of the multifoliate in Chuancao No.7, and aimed to explore the regulatory mechanisms underlying compound leaf development of Alfalfa. These results will provide a theoretical foundation for enhancing the quality and yield of forage.

## 2 Materials and methods

### 2.1 Plant materials and growth conditions

The cultivated alfalfa variety Algonquin was used as a wild-type. In our study, we planted Algonquin in the field and established criteria to screen for plants with multiple leaflets. These criteria included: a percentage of branches with multiple leaflets greater than 90%, a percentage of multiple leaflets exceeding 60%, a minimum of 20 branches per plant, a plant height greater than 70 cm, an upright growth habit, thick stems, and a uniform growth period. After four consecutive years of breeding and screening, a naturally occurring plant with multifoliolate traits was identified. Subsequently, the 2-3 internodes with shoot buds were cut from the multiple leaflet branches and planted in soil in a growth chamber. One month later, the cutting-derived plants were transferred to the field until the seeds matured and were harvested. This process completed a breeding generation referred to as multiple mix selection. After seven generations of screening, the seeds were planted in Xichang, Butuo, and Hanchang in the Sichuan Province of China in 2018 utilizing a randomized block trial design. Each experimental field covered an area of 15 m² (5 m × 3 m) and was surrounded by 1 m wide protective borders. In 2019, all plants from the previous year were propagated asexually through cuttings. Similarly, in 2020, asexual propagation was continued by taking cuttings from the prior year’s all plants. At the early flowering stage, we randomly selected five blocks, each containing ten plants, and assessed the ratio of multiple leaflets among the total leaves of each plant annually in three different experimental fields. The new variety was identified based on a specific criterion of greater than 70% of the multiple leaflets of a single plant and it was licensed as Chuancao No.7 (ChuanS-BV-MS-001-2023) in China.

For *in vitro* plant growth, seeds were scarified with sandpaper and germinated for 4-5 days at 4°C in water containing Petri dishes. Once the roots had germinated to 1cm, the seedlings were transferred to a growth chamber and grown in soil for 4-5 weeks. The growth chamber was at 22°C with 150 μmol m^-2^sec^-1^ light intensity, 16 h day/8 h night photoperiod, and 70 to 80% relative humidity. They were then transferred to the greenhouse under the following conditions: 23°C day/20°C night temperature; 16 h day/8 h night photoperiod; 65% relative humidity and 150 μmol m^-2^sec^-1^ light intensity.

### 2.2 Scanning electron microscopy

Shoot apices were collected from Algonquin and Chuancao No.7 plants 4 weeks after germination. For scanning electron microscopy analysis, plant tissues were fixed overnight in a 3% glutaraldehyde fixative solution (pH 7.0, and 0.1% Trixon-100). The next day, the tissues were washed and dehydrated using a gradient of ethanol. The tissue samples were then dried using liquid carbon dioxide and coated with gold powder. Finally, the samples were examined using a Tecnai Scanning Electron Microscope (SEM) with an accelerating voltage of 5 kV (FEIHillsboro, OS, USA).

### 2.3 Quality evaluation of alfalfa

To assess forage quality, Algonquin and Chuancao No.7 plants with favorable growth status were collected, the above-ground plant biomass was weighed, and the fresh biomass yield was recorded. Five replicates were performed for each sample. Subsequently, the leaf areas of lateral, terminal, and compound leaves were measured, respectively. The leaves of alfalfa plant samples were separated from stems, and the leaf/stem fresh weight ratio was calculated. Leaves and stem samples were oven-dried at 65 °C for 48 h until a stable weight was reached. Then the dried separated leaves and stems were weighed separately, and the leaf/stem dry weight ratio was calculated. For forage quality analysis, plant samples were selected before entering the reproductive growth phase, dried at 105°C for 15 minutes and 60°C-70°C until a stable weight was reached, and ground using an ultra-centrifugal mill with a 1 mm diameter sieve. After grinding the sample powder was sent to the Beijing SauER Environmental Technology Co. LTD for forage quality detection. The NIRS (near infrared reflectance spectroscopy) prediction equations (07AHY50) were employed to calculate the crude protein (CP) (Zhou et al., 2011). Water-soluble sugars (WSS) were determined by a colorimetric method (DuBois et al., 1956). Carbon content was determined using an element analyzer (EA-3000, Boaying Tech., Shanghai, China), while the crude fat (CF) content was assessed according to the GB/T 6433-200 standard (He et al., 2022). Ash content was measured in dry yield by combusting leaves (Horvat et al., 2022). Additionally, both the acid detergent fiber (ADF) content and the neutral detergent fiber (NDF) content were analyzed utilizing the FIWE6 Raw Fiber Extractor (Horvat et al., 2022). Total nitrogen content was calculated using CP content division by 6.25 (Hou et al., 2022). Total digestibility was calculated following the method outlined in Bo et al (Bo et al., 2022). Potassium, phosphorus, calcium, and magnesium content were determined using the AOAC method (Bo et al., 2022). Graphs were generated using Excel 2016. Differences in measured variables of alfalfa were considered at 0.05 and 0.01 probability level was used for the least significant difference (LSD) test.

### 2.4 Transcriptomic analysis

For transcriptomic analysis, 4-week-old alfalfa shoot apices were collected after Algonquin and Chuancao No.7 germination. Three biological replicates of each sample were prepared. Total RNA samples were sequenced using a BGISEQ-500 platform at the Beijing Genomics Institut (BGI, Shenzhen, China). The R package DEGseq was utilized to identify differentially expressed genes (DEGs). DEGs were determined using an up-regulated or down-regulated more than twofold and a false discovery rate (FDR) < 0.01. The hypergeometric test, with *p*-value adjusted using the FDR method, was employed to evaluate the enrichment degree of the Gene Ontology (GO) items and the KEGG pathway.

### 2.5 Protein sequence alignment and phylogenetic analysis

Multiple sequence alignment was performed using Clustal Omega with default parameters. The resulting alignment was used to construct a phylogenetic tree using the neighbor-joining method with 1000 bootstrap replications, as implemented in MEGA6 software (http://www.megasoftware.net/).

### 2.6 RNA extraction and quantitative real-time RT-PCR analysis

The shoot meristem tissue of 4-week-old Algonquin and Chuancao No.7 plants was collected, and total RNA was extracted using TRIzol-RT Reagent (Invitrogen, Carlsbad, CA, USA) according to the manufacturer’s instructions. Total RNA was reverse transcribed into cDNA using the Prime Script II cDNA Synthesis Kit (Takara, Japan) following the manufacturer’s protocol. Subsequently, real-time PCR analysis was performed using Roche SYBR-green reagent (Roche) as the reporter dye on the Bio-Rad CFX Connect TM sequence detection system. Three replicates were performed for each biological sample, and an additional three additional technical replicates were performed for each replicate. Relative expression levels were calculated using the 2^−ΔΔCT^ method. *MsActin* was used as a reference gene. The expression of each gene was normalized to 1 in the Algonquin genotype, and then the expression of the same gene in the Chuancao No.7 genotype was calculated against that. The primers used for qRT-PCR are listed in **Supplementary Table S2**.

### 2.7 Cytokinin treatment

To further investigate the relationship between compound leaf patterns and CK in Algonquin and Chuancao No.7, the following experiments were conducted. Algonquin plants were treated with solutions containing 0 mM, 0.1 mM, 0.25 mM, and 0.5 mM 6-benzyladenine (6-BA) along with 0.01% Tween 20. Based on the preliminary experiment, a concentration of 0.5 mM was selected as the most suitable concentration for the experiment. To inhibit CK activity, different dosages of lovastatin (10 μM, 20 μM, and 40 μM) were employed (Crowell and Salaz, 1992). According to the preparatory experiment, the Algonquin and Chuancao No.7 plants were sprayed with 20 μM lovastatin solution (containing 0.1% DMSO) plus 0.01% Tween 20. The 4-week-old Algonquin shoot apices were treated with 6-BA or lovastatin. Both 6-BA and lovastatin were sprayed in Algonquin and Chuancao No. 7 once daily for 5 consecutive days, and three biological replicates were performed. Plant leaves were taken photos after the treatment for 7 days.

### 2.8 Quantification of endogenous cytokinin

To determine the endogenous CK content of both the wild-type and Chuancao No.7, we collected 4-week-old shoot apices of alfalfa. Three biological replicates were measured per alfalfa line. The 200 mg materials of each replicate were immediately frozen in liquid nitrogen and lyophilized. The samples were then analyzed using an HPLC-MS/MS system (HPLC: ExionLC™ AC; MS: Sciex Triple Quadrupole 4500) by Shandong Guo Cang Jian Biology Co.LTD (Chen et al., 2022). The target material was quantified precisely by isotope internal standard. HPLC analytical conditions were as follows: column was HYPERSIL GOLD C18 column (3μm, 2.1mm*100 mm); solvent A was H_2_O (0.1%FA); solvent B was MeOH; gradient program, 90% A from 0 min to 0.2 min, 90% A at 3 min and kept to 8 min, 10% A at 8.1 min and kept to 10 min; flow rate, 0.3 mL/min; temperature, 35°C; injection volume: 5μL. Triple Quadrupole 4500 HPLC-MS/MS System, equipped with an ESI ion source, was operated in both positive and negative ion mode and controlled by Analyst 1.6.3 software (Sciex). The ESI source operation parameters were as follows: an ion source, ESI+/-; ionspray voltage was 5500V/4500V, source temperature was 550°C; curtain gas (CUR) was set at 30 psi; the collision gas (CAD) was 9 psi. DP and CE for individual MRM transitions were done with further DP and CE optimization. A specific set of MRM transitions was monitored for each period according to the phytohormones eluted within this period.

### 2.9 Statistical analysis

Differences in the measured variables of alfalfa cultivars were evaluated using analysis of variance (ANOVA) with GraphPad Prism version 9.0 software. Student’s t-test was conducted to calculate *p*-values for comparing treatment means. Graphs were created using Excel 2016.

## 3 Results

### 3.1 Compound leaf development process in Algonquin and Chuancao No.7 alfalfa

In 2018, we sowed seeds that were exclusively derived from crosses among the Chuancao No.7 plants. We calculated the percentage of Chuancao No.7 plants relative to the total number of plants across different fields. The results indicated that 97.43% of plants in Xichang, 99.37% in Butuo, and 98.43% in Hanchang displayed multiple leaflets forms. In 2019 and 2020, all plants from the previous year were propagated asexually by cuttings. The ratio of multi-leaflet forms among the total leaves of each plant exceeds 70% annually in three different experimental fields (**Supplementary Table S1**). Based on these data, we conclude that the Chuancao No.7 phenotype is not due to a simple monogenic mutation, but can rather be interpreted as a quantitative trait. This is in accordance with the observations made on a related species, *Trifolium alexandrinum* (Malaviya et al., 2021).

**Figure 1:**
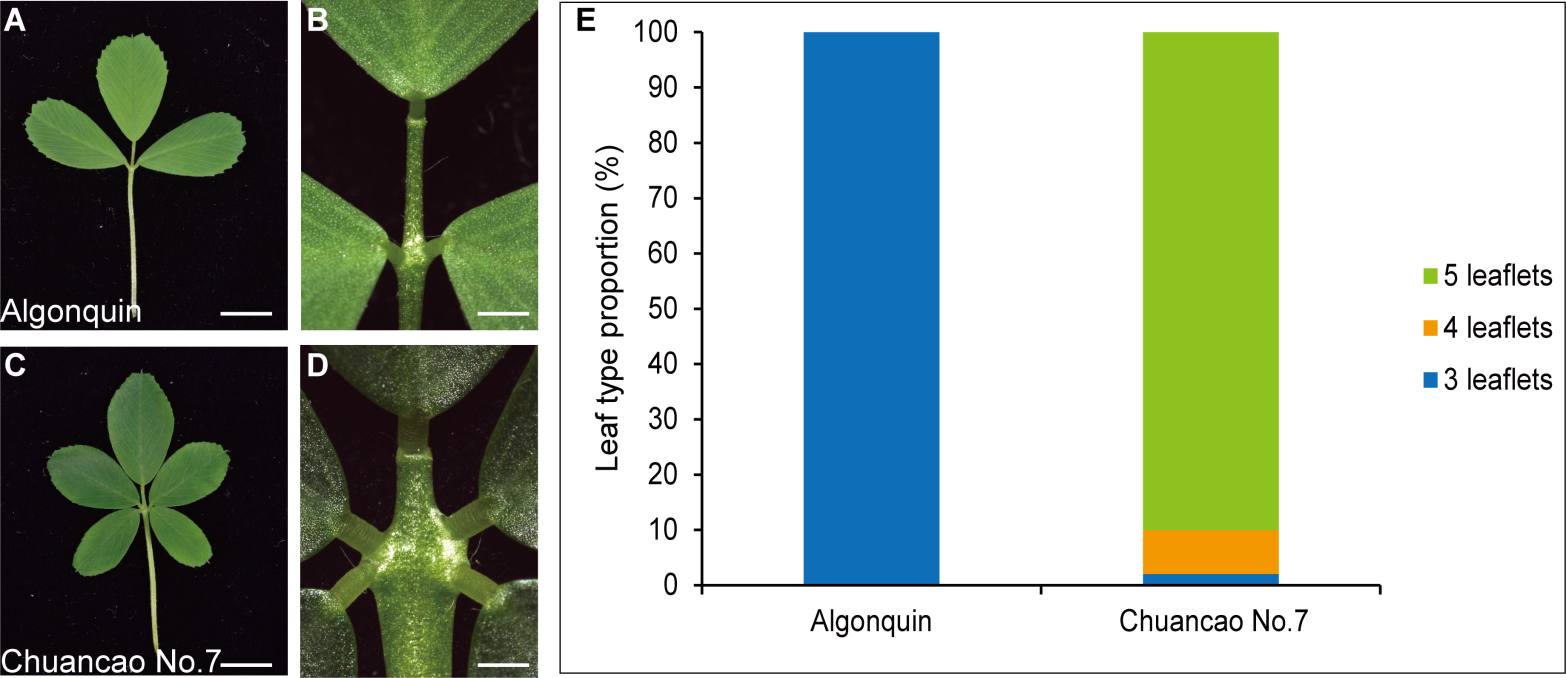
Phenotypic characterization of Algonquin and Chuancao No.7. **(A)** Representative Algonquin compound leaf morphology. **(B)** The close-up view of the leaf region in panel **(A)**. **(C)** Chuancao No.7 compound leaf morphology. **(D)** The close-up view of the leaf region in panel **(C)**. **(E)** The proportion of different compound leaf types among Algonquin and Chuancao No.7. Total compound leaves types were counted from one hundred 4-week-old plants mature compound leaves in the greenhouse for the Algonquin and the Chuancao No.7, respectively. Bars = 0.5 cm **(A, C)**, 1 mm **(B, D)**.

**Figure 2:**
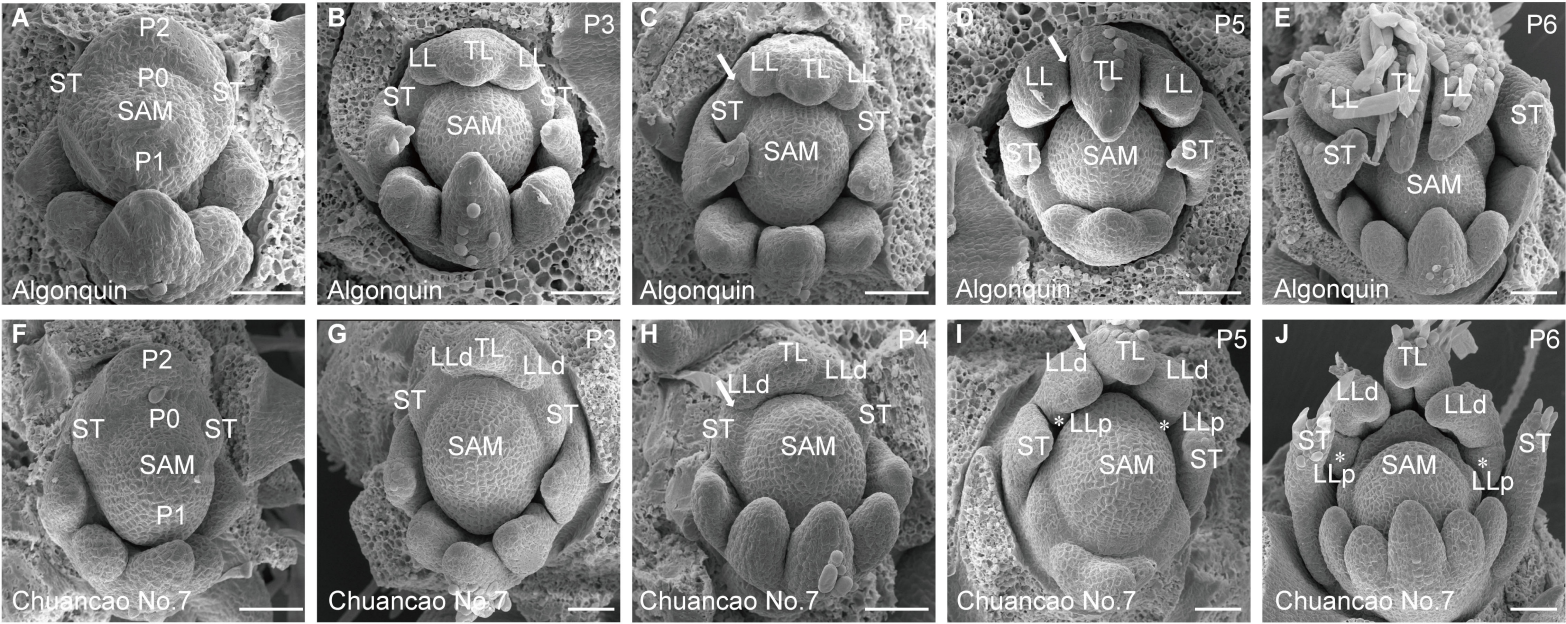
Scanning electron microscopy (SEM) images of Algonquin and Chuancao No.7 leaf primordia at different developmental stages. SEM images of leaf development in Algonquin **(A-E)**, Chuancao No.7 **(F-J)**. **(A, F)** At P0-P2 initiation of leaf primordia development process. During the P0 (Plastochron 0), the initiation of leaf primordia occurred around the SAM **(A)**. The Algonquin initiation of leaf primordia turned to common leaf primordia was emerged from the peripheral zone of the SAM at P1(Plastochron 1). At P2 (Plastochron 2), a pair of stipules primordia (ST) is initiated at the proximal end of the leaf primordium. A pair of stipule primordia (ST) initiated at P2. **(B)** Emergence of lateral leaf primordium (LL) at P3 (Plastochron 3). The common leaf primordium differentiated into a terminal leaflet primordium (TL). **(C)** Boundaries formed between the ST and LL at P4 (Plastochron 4) (arrowhead). **(D)** At P5 (Plastochron 5), boundaries were established between the LL and TL (arrowhead). Trichomes differentiated from the abaxial surface of the TL. **(E)** At P6 (Plastochron 6), the TL became folded and the trichomes were further differentiated into tubular trichomes. **(G)** Emergence of distal lateral leaf primordium (LLd) at P3. **(H)** Boundaries formed between the ST and LLd at P4 (arrowhead). **(I)** At P5, a pair of incipient proximal lateral leaflet primordia (LLp) were formed from the basal of the LLd (asterisk). The arrow indicated the boundary between LLd and TL. **(J)** The LLp was separated away from the LLd (asterisk). Bars = 50 μm **(A-J)**.

The adult leaves of the wild-type Algonquin have a trifoliate form, composed of a terminal leaflet and two lateral leaflets (**Figures 1A, B**). Chuancao No.7 alfalfa showed a substantially increased leaflet (**Figure 1C**), including two pairs of pinnately arranged lateral leaflets and a terminal leaflet at the distal end of the rachis (**Figure 1D**). About 90% of Chuancao No.7 compound leaves have five leaflets type in the greenhouse (**Figure 1E**). Subsequently, we investigated the developmental processes of compound leaves in alfalfa with scanning electron microscopy (SEM). During the P0 (Plastochron 0), the initiation of leaf primordia occurred around the SAM (**Figure 2A**). The Algonquin initiation of leaf primordia turned to common leaf primordia was emerged from the peripheral zone of the SAM at P1(Plastochron 1). At P2 (Plastochron 2), a pair of stipules primordia (ST) is initiated at the proximal end of the leaf primordium. Then, the lateral leaflet primordia (LL) emerged at P3 (Plastochron 3) (**Figure 2B**). Subsequently, at P4 (Plastochron 4) and P5 (Plastochron 5), terminal leaflet primordia (TL), LL, and ST were recognizable (**Figures 2C, D**). Boundaries were formed between the ST and LL, and between the LL and TL (**Figures 2C, D**, arrowhead). At P5, trichomes differentiated from the abaxial surface of the TL. Finally, at P6 (Plastochron 6), the TL became folded and the trichomes further differentiated into tubular trichomes (**Figure 2E**). SEM analyses showed that the leaf development process in Chuancao No.7 alfalfa at the P0-P2 is consistent with that of the Algonquin (**Figure 2F**). Until P3, a pair of incipient distal lateral leaflet primordia (LLd) appeared in the Chuancao No.7 (**Figure 2G**). Boundaries were formed between the ST and LLd at P4 (**Figure 2H**, arrowhead). At P5, two round bulges of cells were formed at the base of the LLd, which are proximal lateral leaflet primordia (LLp) (**Figure 2I**, asterisk). At P6, the LLp was separated away from the LLd (**Figure 2J**, asterisk).

### 3.2 Analysis of forage quality traits

**Figure 3:**
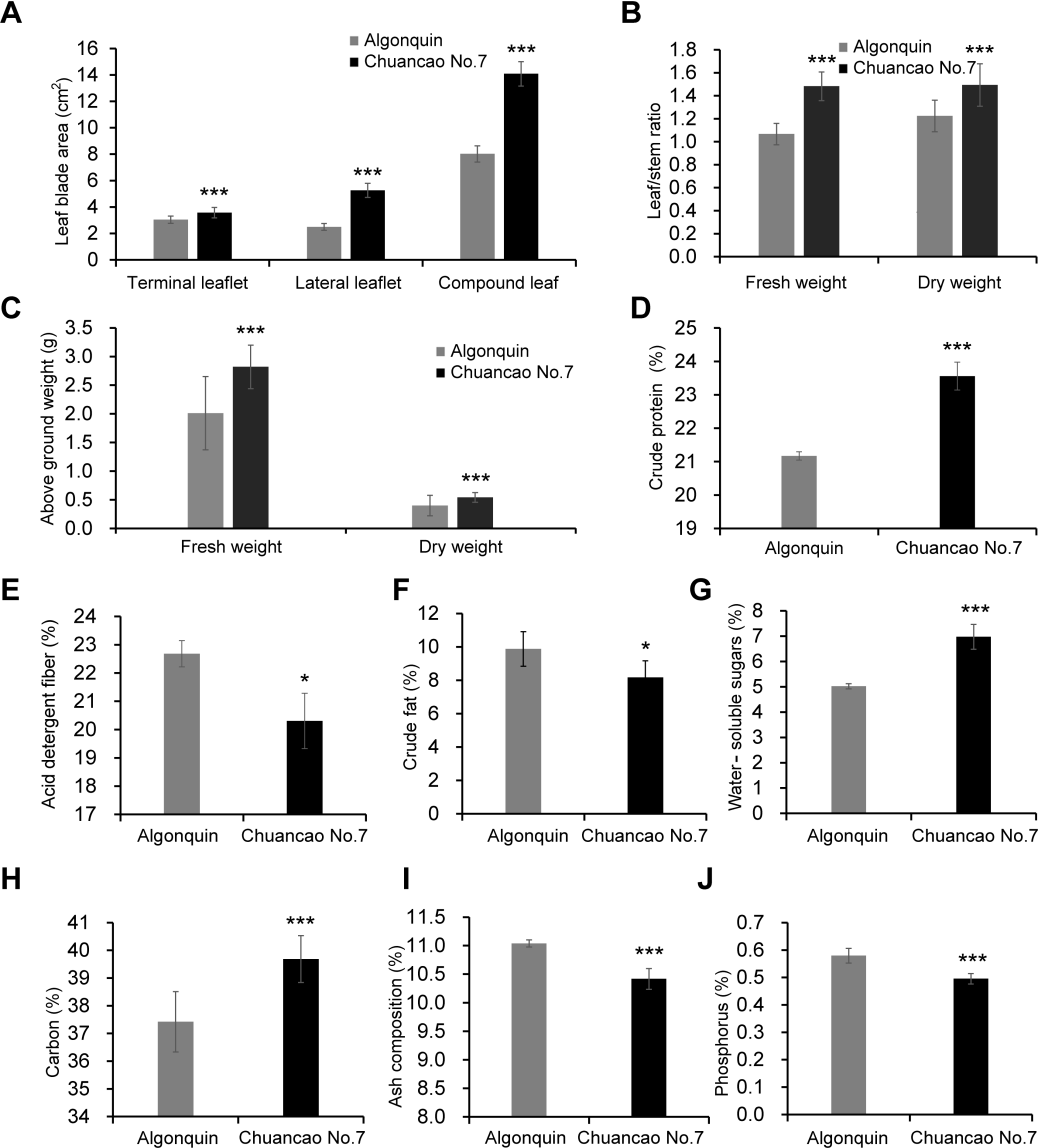
Analysis of forage quality traits. **(A)** Measurements of the blade area of terminal leaflet, lateral leaflet, and compound leaf on Algonquin and Chuancao No.7 alfalfa. **(B)** Measurements of the fresh weight and dry weight of leaf/stem ratio in Algonquin and Chuancao No.7 alfalfa. **(C)** Measurements above the ground weight of Algonquin and Chuancao No.7. **(D-J)** Measurements of the crude protein **(D)**, acid detergent fiber **(E)**, crude fat **(F)**, water-soluble sugars **(G)**, carbon content **(H)**, ash composition content **(I)**, and phosphorus content **(J)** in Algonquin and Chuancao No.7 alfalfa. Values are shown by mean ± SD (n = 3). Values with asterisks (*) are significantly different at 0.01 < *p* ≤ 0.05, and asterisks (***) are significantly different at *p* ≤ 0.001, as analyzed by the student’s t-test.

Compared with the Algonquin, the leaf blade area in terminal leaflets, lateral leaflets, and compound leaf was significantly increased in the Chuancao No.7 (**Figure 3A**). In particular, both lateral leaflets and compound leaf blade areas of the Chuancao No.7 were about twice as large as those of Algonquin. Significant differences in fresh weight and dry weight of the leaf/stem ratios between Algonquin and Chuancao No.7 were detected (**Figure 3B**). Leaf/stem ratios of fresh and dry weights of the Chuancao No.7 were about 1.5 and 1.25 times higher than those in Algonquin. Subsequently, we measured the fresh weight and dry weight of the above-ground parts of the plants in Chuancao No.7 and Algonquin, respectively. The results showed that the fresh weight and dry weight of the Chuancao No.7 were significantly higher than those of the Algonquin (**Figure 3C**). Next, we analyzed the forage quality traits of Algonquin and Chuancao No.7. The key index for evaluating forage quality is CP. As shown in **Figure 3D**, the Chuancao No.7 CP content (23.56%) was significantly higher (*p*-value ≤ 0.001) than that in Algonquin (21.17%). The ADF and CF were lower (0.01 < *p*-value ≤ 0.05) in the Chuancao No.7 compared with the Algonquin (**Figures 3E, F**). Both the WSS content and the carbon content were increased significantly (*p*-value ≤ 0.001) in the Chuancao No.7 than in Algonquin (**Figures 3G, H**). However, the ash composition content and phosphorus content in Chuancao No.7 alfalfa were significantly lower (*p*-value ≤ 0.001) than that of the Algonquin (**Figures 3I, J**). However, there were no significant differences (*p*-value > 0.05) in other traits between the Chuancao No.7 and Algonquin, including the nitrogen content, potassium content, neutral detergent fiber (NDF) content, digestibility, calcium content, and magnesium content (**Supplementary Figure S1**).

### 3.3 Transcriptomic analysis of Algonquin and Chuancao No.7

**Figure 4:**
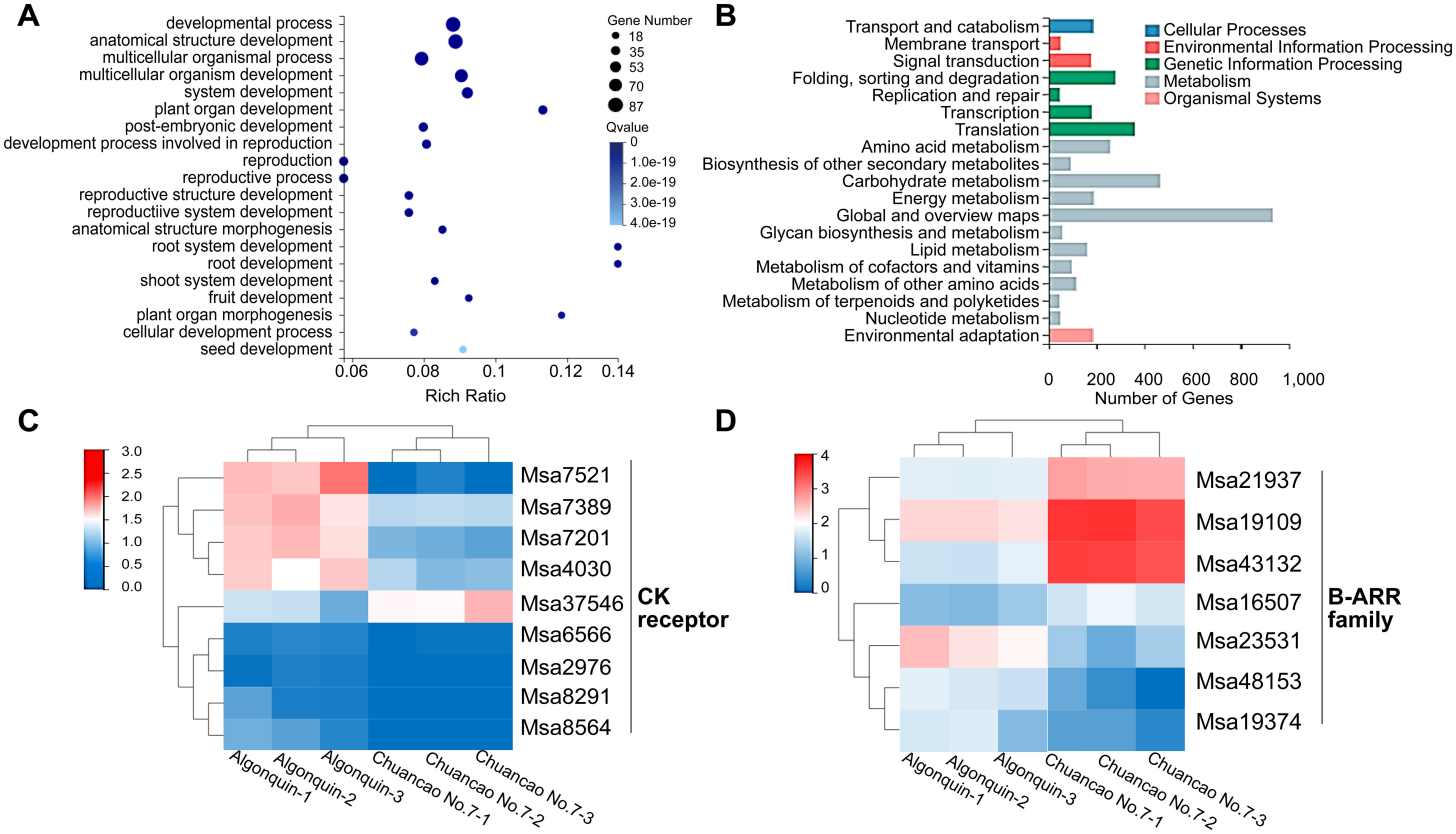
Transcriptomic of Algonquin and Chuancao No.7. **(A)** GO rich of differentially expressed genes during compound leaf development of Algonquin and Chuancao No.7. **(B)** KEGG classification of differentially expressed genes during compound leaf development of Algonquin and Chuancao No.7. **(C)** Heatmap representation of relative expression values of cytokinin receptor differentially expressed genes of Algonquin and Chuancao No.7. **(D)** Heatmap representation of relative expression values of B-ARR family differentially expressed genes between Algonquin and Chuancao No.7.

To further explore the leaf developmental mechanism of the Chuancao No.7 alfalfa, we utilized RNA-seq to characterize the transcriptome of 4-week-old Algonquin and Chuancao No.7 plants. A total of 4, 926 different DEGs were identified, including 1, 859 up-regulated and 3, 067 down-regulated genes (**Supplementary Figure S2A**; **Supplementary Table S3**). A higher correlation coefficient indicated greater similarity of gene expression levels in different samples (**Supplementary Figure S2B**). To further explore the function of DEGs between Algonquin and Chuancao No.7, we used KEGG and GO annotations to analyze the main biological functions of DEGs (**Supplementary Tables S4**, **S5**). Among the significant GO enrichment annotations, the development process, anatomical structure development, and multicellular organismal process contained more genes than the others (**Figure 4A**). DEGs were enriched in multicellular organismal processes implying that the multicellular formation might have a modulating effect on the process of Chuancao No.7 alfalfa leaf development. Multicellular organismal development was consistent with the process of rapid cell division of SAM and leaf morphogenesis. The KEGG pathway analysis showed that DEGs were classified into several categories: cellular processes, environmental information processing, genetic information processing, metabolism, and organismal systems (**Figure 4B**). Signal transduction in environmental information processing is an important step of plant development, which includes the plant hormone signal transduction pathway.

In the transcriptomes of the CK signaling pathway, 9 DEGs in the CK receptor, and 7 DEGs in the type-B ARR family were enriched (**Figures 4C, D**; **Supplementary Table S6**). The expression levels of CK receptor gene *Msa37546* and type-B ARR genes *Msa21937*, *Msa19109*, *Msa43132*, and *Msa16507* were higher in the Chuancao No.7 than in the Algonquin, indicating that these CK signal transduction genes are probably involved in the leaf development of Chuancao No.7 alfalfa.

We also identified 9 MsKNOXI proteins according to the phylogenetic analysis of KNOXI protein from *M. truncatula* and *M. sativa* (**Supplementary Figure S3A**). To investigate whether *MsKNOXI* genes are involved in the leaf development of alfalfa, qRT-PCR experiments were performed (**Supplementary Figure S3B**). The results showed that the expression of *MsKNOX2* was upregulated higher than other genes in Chuancao No.7 alfalfa.

### 3.4 Cytokinin plays a key role in the alfalfa compound leaf development

**Figure 5:**
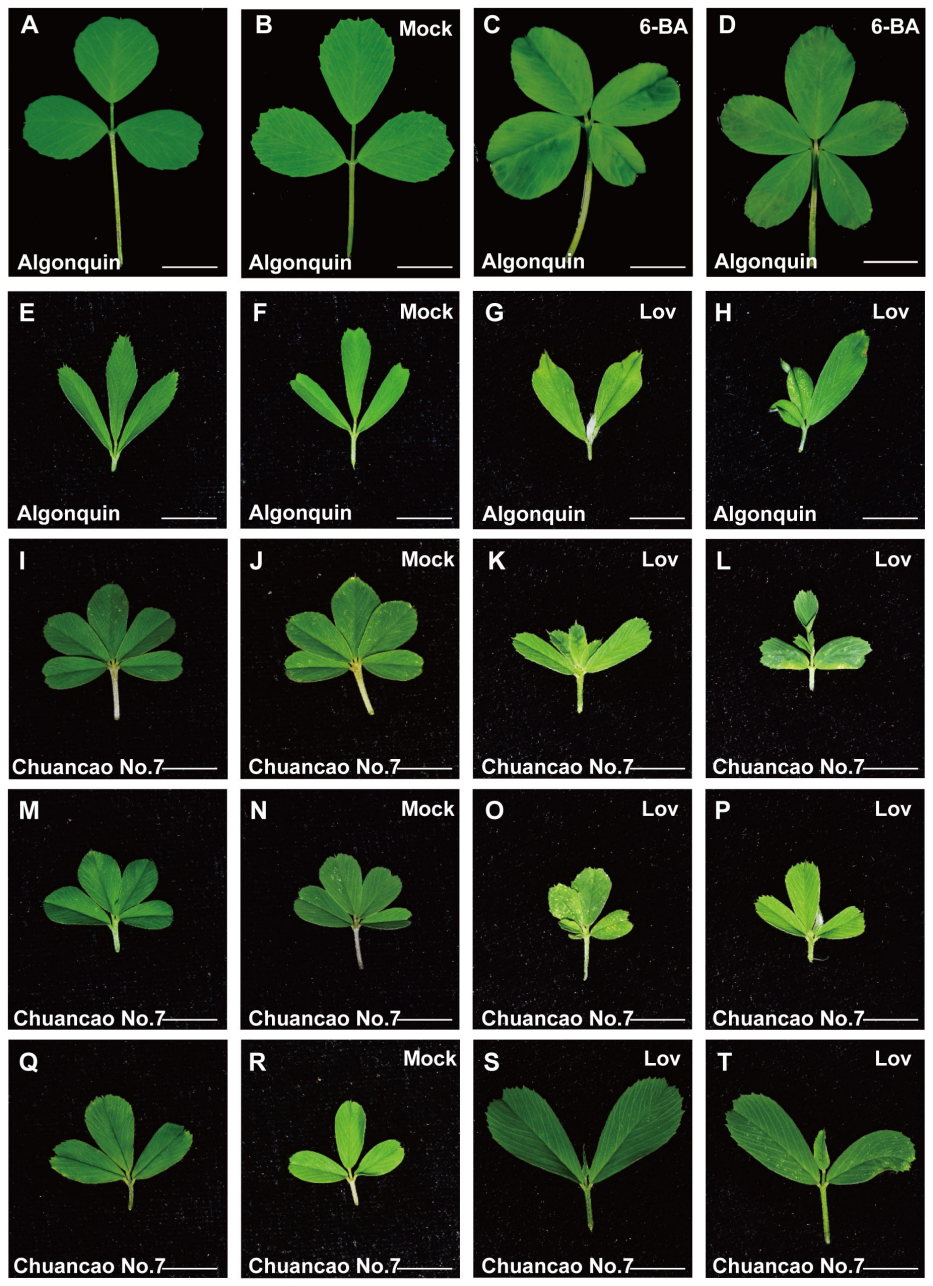
Effect of 6-BA and lovastatin (lov, CK inhibitor) treatment of Algonquin and Chuancao No.7. **(A)** Adult leaf of Algonquin. **(B)** Adult leaf of Algonquin treated with the Tween 20 as control. **(C, D)** Adult leaves of Algonquin treated with 0.5 mM 6-benzyladenine (6-BA) plus 0.01% Tween 20. **(E)** Young leaf of Algonquin. **(F)** Leaf of Algonquin treated with Tween 20 as control after 7 days. **(G, H)** Algonquin leaves were treated after 7 days with 20 μM lovastatin solution (containing 0.1% DMSO) plus 0.01% Tween 20. **(I)** The Chuancao No.7 has a compound leaf type with five leaflets. **(J)** Five leaflets of Chuancao No.7 were treated with Tween 20 as control after 7 days during growth. **(K, L)** Chuancao No.7 was treated with 20 μM lovastatin solution (containing 0.1% DMSO) plus 0.01% Tween 20. **(M)** The Chuancao No.7 has a compound leaf type with four leaflets. **(N)** Four leaflets of Chuancao No.7 were treated with Tween 20 as control after 7 days during growth. **(O, P)** Chuancao No.7’s four leaflets were treated with 20 μM lovastatin solution (containing 0.1% DMSO) plus 0.01% Tween 20. **(Q)** The leaf of the Chuancao No.7 has a trifoliate leaf type. **(R)** Three leaflets of Chuancao No.7 were treated with Tween 20 as control after 7 days during growth. **(S, T)** Chuancao No.7 trifoliate leaflets were treated with 20 μM lovastatin solution (containing 0.1% DMSO) plus 0.01% Tween 20. Mock, Leaf treated with 0.01% Tween 20 as control. Bars = 1 cm **(A-J)**.

The adult leaves of the Algonquin have a trifoliate form (**Figure 5A**), and the Tween 20 was treated as control (**Figure 5B**). The results showed that 0.5 mM 6-BA treatment could induce the additional leaflets between two lateral leaflets, and the leaf complexity was increased (**Figures 5C, D**). Some of the Algonquin leaves were changed from trifoliate alfalfa to a pentafoliate form, which mimicked the Chuancao No.7 phenotype. Furthermore, the SAM of Algonquin and Chuancao No.7 were treated with 20 μM lovastatin for 7 days, a specific inhibitor of CK biosynthesis. The lovastatin treatment inhibited the leaf development of Algonquin, compared with the controls (**Figures 5E–H**). Similar results were observed in Chuancao No.7 when they were treated with lovastatin. As seen in **Figures 5I–T**, under normal conditions without lovastatin, the Chuancao No.7 has three compound leaf types containing five leaflets (**Figure 5I**), four leaflets, and a trifoliate leaf pattern (**Figures 5M, Q**). The same concentration of Tween 20 treatment did not alter leaf morphology and leaflet number in the Chuancao No.7 (**Figures 5J, N, R**). When the Chuancao No.7 was sprayed with lovastatin, the terminal leaflet and distal lateral leaflet became smaller or even disappeared (**Figures 5K, L**). Some of the Chuancao No.7 with four leaflets showed the delayed development of lateral leaflets (**Figures 5O, P**). The trifoliate leaf pattern of the Chuancao No.7 was consistent with those of the Algonquin after lovastatin treatment (**Figures 5S, T**).

**Figure 6:**
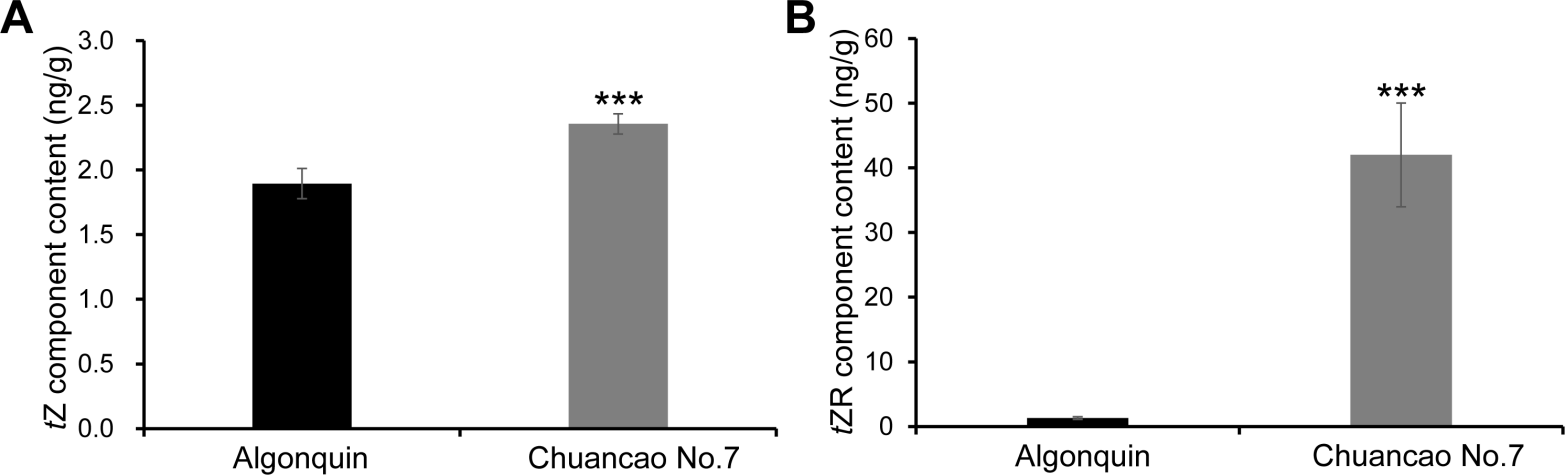
Cytokinin content in the Chuancao No.7 and Algonquin. **(A)** *Trans*-zeatin (*t*Z) component content in the Chuancao No.7 and Algonquin. **(B)** *Trans*-zeatin riboside (*t*ZR) component content in the Chuancao No.7 and Algonquin. Values are shown by mean ± SD (n = 3). Values with asterisks (***) are significantly different at *p* ≤ 0.001, as analyzed by the student’s t-test.

The CKs are naturally occurring hormones, *trans*-zeatin (*t*Z) and *trans*-zeatin riboside (*t*ZR) type CKs are the major forms in crops (Sakakibara, 2006). Therefore, we evaluated the endogenous *t*Z and *t*ZR content of both the Algonquin and Chuancao No.7 alfalfa. As the results showed, the *t*Z and *t*ZR content of Chuancao No.7 plants were both higher than Algonquin (**Figures 6A, B**). In particular, Chuancao No.7 *t*ZR content was almost 40 times as much as that of Algonquin. These results indicated that the increased CK content was sufficient to promote alfalfa leaflet development. CK is responsible for the increased number of Chuancao No.7 leaflets.

## 4 Discussion and conclusions

### 4.1 Significance of improving forage quality of Chuancao No.7 alfalfa leaves

Investigating agronomic traits comprehensively allows for a better understanding of the growth and morphological characteristics of plants, aiding in the identification of an excellent variety of resources and serving as the basis for the evaluation of forage quality. In addition, The leaf/stem ratio, fresh yield, and dry yield serve as crucial indicators for assessing forage palatability and influence forage production (Tucak et al., 2014). In this study, the leaf blade area, leaf/stem ratio, total fresh weight, and dry weight had significantly increased in Chuancao No.7 alfalfa compared to Algonquin (**Figures 3A–C**). Therefore, the Chuancao No.7 alfalfa exhibited a higher leaf/stem ratio, fresh yield, and dry yield, making it suitable for improving new lines for high yield.

Alfalfa leaves are rich in highly digestible proteins, which are one of the most important indicators for evaluating forage nutrition quality. In this study there was a highly significant difference in CP content between the two alfalfa varieties, and the crude CP content of the Chuancao No.7 was 23.56%, indicating agreement with the criteria for good forage quality established by American Forage and Grassland Council USDA (**Figure 3D**) (Rohweder et al., 1978). Fiber content is inversely related to forage quality; lower fiber indicates higher digestibility and a greater feeding value of forage (Jungers et al., 2020). We found that ADF content decreased significantly by about 2% in the Chuancao No.7, indicating that Chuancao No.7 alfalfa is easier to eat and digest by ruminants compared to the Algonquin (**Figure 3E**). The CF content of different types of alfalfa varies constantly under the same cultivation conditions (Horvat et al., 2022). The CF content of the Chuancao No.7 was significantly lower than that of the Algonquin, which may account for the variation in alfalfa varieties (**Figure 3F**). The Chuancao No.7 may enhance livestock digestibility and utilization due to the high content of water-soluble sugars compared to Algonquin (**Figure 3G**). Owing to its varied leaf morphology, Chuancao No.7 has a higher total carbon content than Algonquin (**Figure 3H**), representing it will provide more fundamental material for Chuancao No.7 growth and development. Ash composition is not a nutrient in feed; therefore the lower its content, the higher its nutritional value (Feng et al., 2023). As we expected, the ash composition content of the Chuancao No.7 was lower than that of the Algonquin (**Figure 3I**). Excessively high or low phosphorus content is not conducive to the normal growth of alfalfa (Peng et al., 2021). The phosphorus content should remain within the normal range as the plant biomass was not affected in Algonquin and Chuancao No.7 plants. This study investigated the nitrogen, potassium, calcium, and magnesium content and found that both the Chuancao No.7 and Algonquin exhibited strong nitrogen-fixing abilities and superior nutrient and mineral content (**Supplementary Figures S1A, C**). In conclusion, the forage quality of the Chuancao No.7 alfalfa surpassed that of Algonquin. Chuancao No.7 could be employed in alfalfa breeding programs to develop elite transgene-free alfalfa cultivars.

### 4.2 Relationship between CK and compound leaf development

Alfalfa is extensively cultivated as a legume for livestock, characterized by a typical trifoliate leaf pattern, with 70% of its protein stored within the leaves (Hojilla-Evangelista et al., 2017). Nonetheless, leaf patterns exhibit significant variability among different alfalfa varieties. In this study, we investigated a naturally occurring Chuancao No.7 alfalfa variant characterized by additional leaflets, resulting in the transformation of the compound leaf pattern from trifoliate to pentafoliate form. Transcriptomic and KEGG pathway analyses of Algonquin and Chuancao No.7 alfalfa with additional leaflets were conducted to elucidate the molecular mechanism underlying the modulated leaf development, particularly in multifoliate forms (pinnate pentafoliate type). The results suggested that CK signal transduction significantly influenced leaf development by playing a central role in the cell cycle and impacting leaf growth.

CK signal transduction plays a crucial role in regulating leaf development. The CK-deficient *Arabidopsis* will develop with smaller apical meristems and leaf cell production was only 3-4% of that of wild-type, indicating an absolute requirement of CK for leaf development (Werner et al., 2001). CK signal transduction involves a multistep phosphorelay system known as a two-component model (Zwack and Rashotte, 2014). According to the gene annotation, the CK receptor gene *Msa37546* is the homologous gene of *AHKs* in *Arabidopsis*, while *Msa21937*, *Msa19109*, *Msa43132*, and *Msa16507* are the homologous genes of type-B ARRs genes family. Mutants of the *Arabidopsis ahk2*,*3*,*4* receptor exhibits a reduction in SAM size ​and severe growth retardation (Nishimura et al., 2004). However, the majority of single mutants in the CK signaling pathway do not exhibit distinct morphological phenotypes. Hence, we hypothesized that *Msa37546* might interact with other genes in the CK pathway to regulate alfalfa leaf development. In *Arabidopsis*, there are two types of response regulators: type-B ARRs, which are activated by phosphorylation and mediate CK-regulated gene expression, and type-A ARRs, which function as negative feedback elements in CK signaling (Cortleven et al., 2019). There are various type-B ARRs in the *Arabidopsis* genome, possessing a transcription factor domain, that could regulate plant response or output in the perception of CK. As positive regulators of CK signal, the major type-B RR family exhibits largely redundant functions. Their immediate transcription targets are type-A RRs, which mitigate the output in response to elevated CK levels (Mason et al., 2005). Some of the type-B ARR genes were upregulated in the Chuancao No.7 alfalfa, suggesting their likely role as positive regulators of the CK signaling pathway in alfalfa.

Cytokinins play an essential role in maintaining the growth of shoot apical meristems, which furnish stem cells for the formation of leaf primordia (Kalve et al., 2014). The study results suggest CK is indispensable for alfalfa leaf development and can stimulate leaflet cell proliferation when treated with 6-BA. However, when cytokinin biosynthesis is inhibited, alfalfa leaves display a phenotype characterized by restricted cell proliferation and decreased number of leaflets. *Trans*-zeatin (*t*Z) is recognized as a biologically active compound that is central to cytokinin functions (Schaller et al., 2015). *Trans*-zeatin riboside (*t*ZR) is utilized for the direct initiation of shoot apical cells and promotes the sprouting of lateral buds (Kuroha, 2002). Analysis of endogenous cytokinin content revealed that levels of *t*Z and *t*ZR were significantly increased in the Chuancao No.7. In the Chuancao No.7 plant, a slight increase in *t*Z and a dramatic increase in *t*ZR are likely the result of both enhanced CK biosynthesis and the concurrent ribosylation of free *t*Z into the inactive *t*ZR form. Most likely, *t*Z is responsible for the compound leaf formation.

*KNOXI* genes can activate the CK pathway to regulate leaf development. In previous studies, CK level is positively regulated by *KNOXI* family genes, such as *SHOOT-MERISTEMLESS* (*STM*), *KNOTTED-LIKE FROM ARABIDOPSIS THALIANA 1* (*KNAT1*), *KNAT2*, and *KNAT6* to maintain meristem development (Wu et al., 2021). In addition, the overproduction of endogenous cytokinin significantly increases the levels of the *KNOX I* genes in *Arabidopsis*, forming a positive feedback loop between *KNOX I* genes and cytokinins (Rupp et al., 2002). In *M. truncatula*, *MtKNOXI* was observed at the early stages of leaf primordia formation, and overexpression of *MtKNOXI* genes is sufficient to increase leaf complexity (Di Giacomo et al., 2008; Lu et al., 2024). According to this research, qRT-PCR experiments were performed and the expression of *MsKNOX2* was upregulated in the Chuancao No.7, indicating that it may regulate the leaf development of alfalfa. *MsKNOX2* may increase cytokinin levels to diversify the compound leaf pattern in alfalfa.

In this study, a Chuancao No.7 alfalfa was derived from the wild-type of Algonquin with multiple leaflets phenotype. The Chuancao No.7 plants exhibit excellent agronomic and forage quality traits. Investigation of these Chuancao No.7 populations over a long period is necessary to fully understand their superior traits because alfalfa is a cross-pollinated species with complex genomics. We look forward to using quantitative trait loci (QTL) analysis between Chuancao No.7 and wild-type, to screen and map genes related to compound leaf development of alfalfa. Meanwhile, it is also an important research direction to more deeply explore the function of cytokinin synthesis and signal transduction genes related to leaf development in Chuancao No.7 alfalfa.

## Data Availability

Data sets generated during this study are available at the National Center for Biotechnology Information (NCBI) with BioProject accession number PRJNA1116326.
