# Supplementary material for: Transcriptomic and physiological analyses reveal that cytokinin is involved in the compound leaf development of alfalfa
Source: Front Plant Sci. 2025 Jan 29;16:1460205. doi: 10.3389/fpls.2025.1460205 (PMC11814202; doi:10.3389/fpls.2025.1460205)
Supplement: Supplementary file 1 [file DataSheet1.docx]

Supplementary Material

**Supplementary Table S1** The ratio of multiple leaflets of each Chuancao No.7 plant

| Experimental field | 2018 year (%) | 2019 year (%) | 2020 year (%) |
| --- | --- | --- | --- |
| Xichang | 72.39 ± 1.03 | 71.24 ± 6.21 | 73.28 ± 11.15 |
| Butuo | 72.25 ± 4.81 | 71.80 ± 1.67 | 70.68 ± 4.22 |
| Hanchang | 72.86 ± 1.93 | 70.94 ± 2.75 | 70.19 ± 4.78 |

Note: Values are shown by mean ± SD (n = 50).


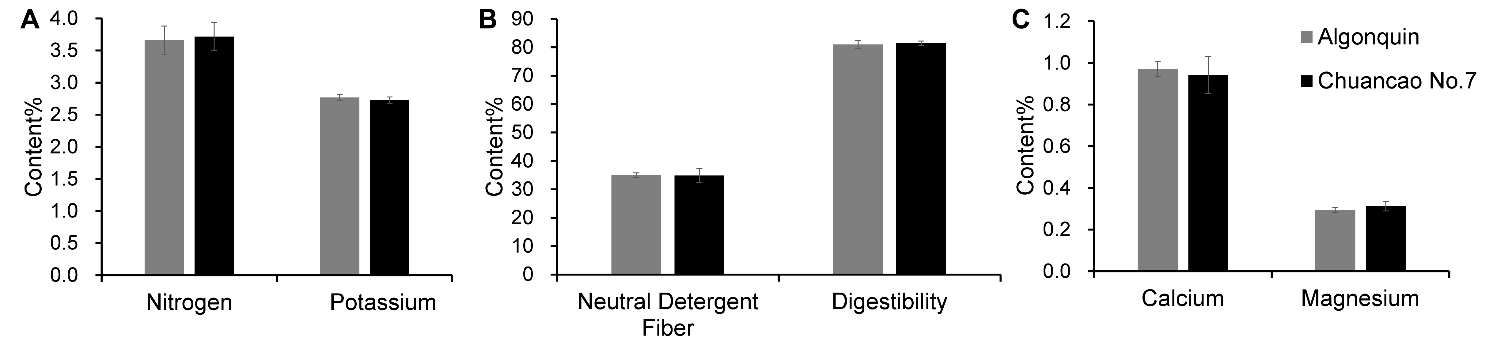


**Supplementary Figure 1. Analysis of forage quality traits.** **(A)** Measurements of the nitrogen content and potassium content of Algonquin and Chuancao No.7 alfalfa. **(B)** Measurements of the neutral detergent fiber (NDF) content and digestibility of Algonquin and Chuancao No.7 alfalfa. **(C)** Measurements of the calcium content and magnesium content of Algonquin and Chuancao No.7 alfalfa. Values are shown by mean ± SD (n = 3).


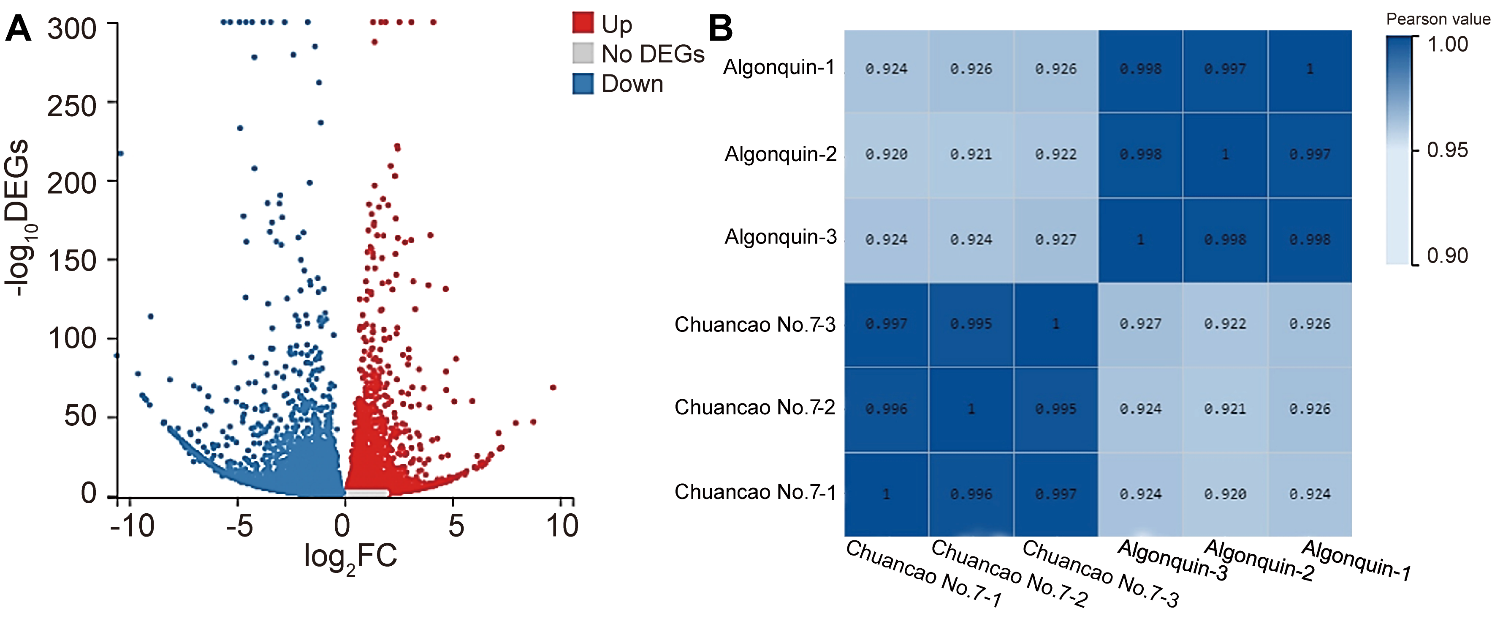


**Supplementary Figure 2.** Volcano map and correlation heat map of differentially expressed genes in Algonquin and Chuancao No.7. **(A)** Volcano map of differentially expressed genes (DEGs) in Algonquin and Chuancao No.7 alfalfa. The X-axis represents the multiple differences in each group, and the Y-axis represents the number of DEGs. Red dots represent up-regulated genes, blue dots represent down-regulated genes, and gray dots represent unchanged genes. **(B)** Pearson value can reflect the correlation coefficient of gene expression between each sample. The higher the correlation coefficient, the higher the similarity of gene expression level.


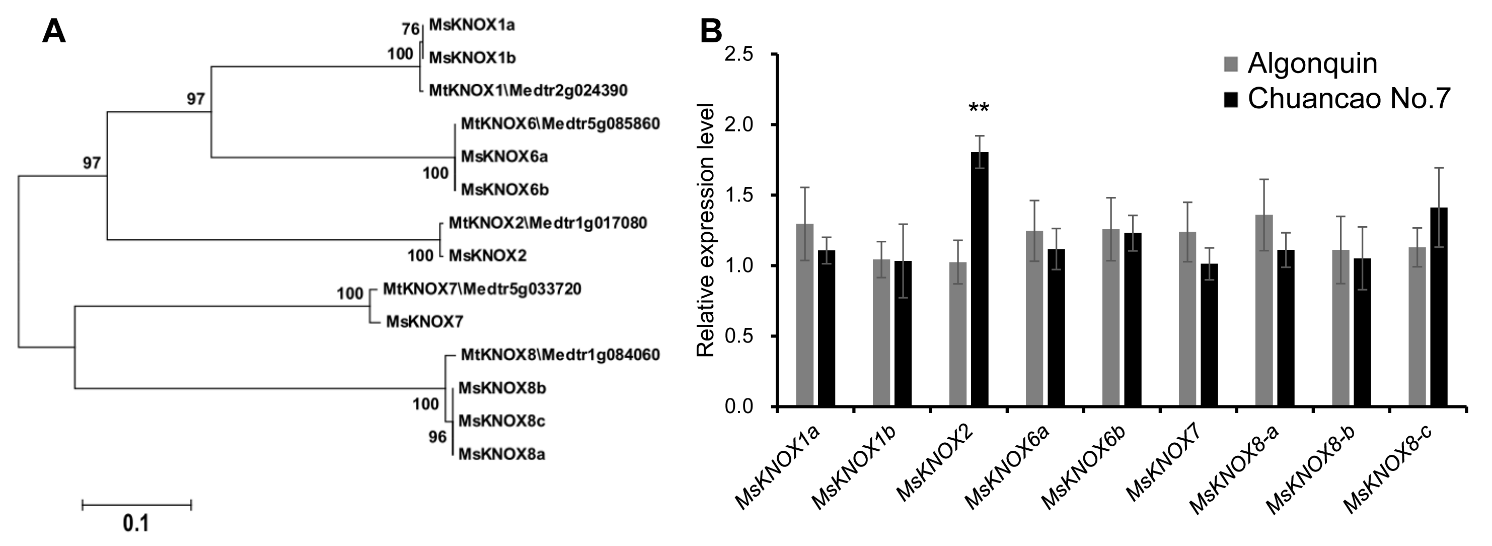


**Supplementary Figure 3.** Expression analysis of *MsKNOXⅠ* related to compound leaf development. **(A)** Phylogenetic tree of KNOXⅠ proteins from *M. truncatula* and *M. sativa*. Mt, *Medicago truncatula*. Ms, *Medicago sativa*. **(B)** The expression level of *MsKNOXⅠ* related to compound leaf development in Algonquin and Chuancao No.7. Values are shown by mean ± SD (n = 3). Values with asterisks (**) are significantly different at *p* ≤ 0.01, as analyzed by the student’s t-test.
